# Another Danger Signal out

**Published:** 1902-08

**Authors:** 


### ANOTHER DANGER SIGNAL OUT.

At last the field of veterinary science has been invaded under the auspices of a correspondence school. Its advent is announced from Iowa, and no doubt will be followed by like institutions in other States to gull money out of easily deluded fools over the land.

It is extremely unfortunate that it should have its birth through the announced promotion of one of the members of the Iowa State Veterinary Medical Association, and that body should most summarily deal with the offender by expelling him from the Association.

Iowa has never done better work for the true growth of the profession than during the past five years. The college at Ames has been materially strengthened in her teaching staff, and more financial aid to this department is expected in the near future. The State Association is one of the most active and progressive in the land, the officers and members are all active in good work, and it was the last field in which to look for the development of such a dangerous institution. The seductive letters sent out by the head of this correspondence school are dangerous, indeed, and the commercial aspect of the whole movement is written between the lines as plainly as the noonday sun. It should, in the language of the Scotchman, "be scotched," and we are sure our Iowa friends will lead in the effort.

